# Analysis of Sequence Variation and Risk Association of Human Papillomavirus 52 Variants Circulating in Korea

**DOI:** 10.1371/journal.pone.0168178

**Published:** 2016-12-15

**Authors:** Youn Jin Choi, Eun Young Ki, Chuqing Zhang, Wendy C. S. Ho, Sung-Jong Lee, Min Jin Jeong, Paul K. S. Chan, Jong Sup Park

**Affiliations:** 1 Departments of Obstetrics and Gynecology, Seoul St. Mary’s Hospital, College of Medicine, The Catholic University of Korea, Seoul, Republic of Korea; 2 Department of Microbiology, Faculty of Medicine, The Chinese University of Hong Kong, Hong Special Administrative Region, China; Georgetown University, UNITED STATES

## Abstract

**Introduction:**

Human papillomavirus (HPV) 52 is a carcinogenic, high-risk genotype frequently detected in cervical cancer cases from East Asia, including Korea.

**Materials and Methods:**

Sequences of HPV52 detected in 91 cervical samples collected from women attending Seoul St. Mary’s Hospital were analyzed. HPV52 genomic sequences were obtained by polymerase chain reaction (PCR)-based sequencing and analyzed using Seq-Scape software, and phylogenetic trees were constructed using MEGA6 software.

**Results:**

Of the 91 cervical samples, 40 were normal, 22 were low-grade lesions, 21 were high-grade lesions and 7 were squamous cell carcinomas. Four HPV52 variant lineages (A, B, C and D) were identified. Lineage B was the most frequently detected lineage, followed by lineage C. By analyzing the two most frequently detected lineages (B and C), we found that distinct variations existed in each lineage. We also found that a lineage B-specific mutation K93R (A379G) was associated with an increased risk of cervical neoplasia.

**Conclusions:**

To our knowledge, we are the first to reveal the predominance of the HPV52 lineages, B and C, in Korea. We also found these lineages harbored distinct genetic alterations that may affect oncogenicity. Our findings increase our understanding on the heterogeneity of HPV52 variants, and may be useful for the development of new diagnostic assays and therapeutic vaccines.

## Introduction

Human papillomavirus (HPV) is the major causative agent of cervical cancer, a leading cause of death among women worldwide [1]. The virus genome is divided into three functional regions: an upstream regulatory region, an early region, and a late region. The upstream regulatory region is a non-coding region, referred as the long control region (LCR), which regulates transcriptional and replication activities. In comparison, the early and late regions are coding regions. Early regions (E1, E2, E4, E5, E6 and E7) encode for non-structural proteins while late regions (L1 and L2) encode for structural proteins [2]. HPV is markedly heterogeneous with more than 200 genotypes which are classified into types, lineages, and sub-lineages based on the L1 sequence. The L1 sequences among different types differ by at least 10%, and those of lineages differ by >1% [3, 4]. The persistence of HPV contributes to the progression of cervical infection to cervical cancer. In particular, oncogenicity varies according to the HPV genotype, as well as the lineage of some genotypes [4–6]. Eight most common high-risk HPV genotypes (HPV16, HPV18, HPV31, HPV33, HPV35, HPV45, HPV52 and HPV58) are responsible for 91% of cervical cancers [7, 8]. Of these, HPV52 is a high-risk genotype and one of the nine HPV types (HPV6, HPV11, HPV16, HPV18, HPV31, HPV33, HPV45, HPV52 and HPV58) targeted by the recent Food and Drug Administration (FDA) approved HPV 9-valent vaccine [9].

Given that HPV52 is recognized as a high-risk genotype commonly found in cervical cancers from East Asia [10–12], we attempted to characterize HPV52 variants circulating in Korea and to investigate their association with cervical cancer development.

## Materials and Methods

### Cervical samples

Altogether, 91 cervical cytology/tissue samples that had tested positive for HPV52 were used for this study. These samples had been collected as part of the routine clinical management at Seoul St. Mary’s Hospital (Seoul, Korea) and all were treatment-naive. This study was approved by the institutional review board of the Catholic University of Korea, College of Medicine and the participants provided written informed consent. Pathologic features of patients are summarized in Table 1. The quality of DNA extracted from cytology/tissue samples was assessed by amplifying a 932-bp fragment of the long-control region (LCR). The HPV genotype was ascertained by demonstrating a nucleotide sequence similarity of >90%, compared with the HPV52 prototype (GenBank accession no. X74481).

**Table 1:** Cervical pathologies of study subject.

Cervical pathology		Number of subjects (percentage) (n = 91)	Lineages				Mean age (years) (±SD)
			A (n = 5)	B (n = 79)	C n = 6)	D (n = 1)	
Normal		40 (44.0)	3	35	2	0	40.2 (±9.6)
Low-grade lesions		22 (24.2)	2	19	0	1	39.6 (±10.7)
	ASCUS	2					
	LGSIL	20					
High-grade lesions		21 (23.1)	0	17	4	0	46.7 (±11.9)
	HGSIL	14					
	CIN3	4					
	CIS	3					
	SCC	7 (7.7)	0	7	0	0	57.6 (±18.0)
Unknown		1 (1.1)	0	1	0	0	

ASCUS: atypical squamous cells of undetermined significance; CIN3: cervical intraepithelial neoplasia 3; CIS: carcinoma *in situ*; LGSIL: low-grade squamous intraepithelial lesions; HGSIL: high-grade squamous intraepithelial lesions; SCC: squamous cell carcinoma.

### Nucleotide sequencing

E6, E7, L1 and LCR sequences were amplified with long- or short-fragment polymerase chain reaction (PCR). Long-fragment PCR was performed on good-quality samples with primers 5′-ATG TCC ATT GAG TCA GGT CC-3′ and 5′-TGC ATT TTC ATC CTC GTC C-3′. When the first-round PCR product was not strong enough for sequencing, a second-round PCR was performed using inner primers 5′-GGT CCT GAC ATT CCA TTA CC-3′ and 5′-CCT CTA CTT CAA ACC AGC CT-3′ when necessary ′ (S1 Table). Each PCR was conducted in a 50-μL reaction mixture containing 1 unit of Phusion Hot Start II High-Fidelity DNA Polymerase (Thermo Fisher Scientific, Waltham, USA), 200 μM of dNTPs and 0.25 μM of each primer. An aliquot of 5 μL of extracted DNA was added as template. The thermal cycling began with a 30-sec initial denaturation and enzyme activation at 98°C, followed by 35 cycles of 10-sec denaturation at 98°C, 30-sec annealing at 62°C and 100-sec extension at 72°C, and ended with an 10-min final extension at 72°C. When long-fragment PCR was not successful, short-fragment PCR was performed with primer pairs E6/E7 (5′-TGC ACT ACA CGA CCG GTT A-3′ and 5′-CAT CCT CGT CCT CTG AAA TG-3′), L1A (5′-ATG TCC ATT GAG TCA GGT CC-3′ and 5′-GCA CAG GGT CAC CTA AGG TA-3′), L1B (5′-AGG ATG GGG ACA TGG TAG AT-3′ and 5′-CAC AGA CAA TTA CCC AAC AGA C-3′) and LCR (5′-GTC TGC ATC TTT GGA GGA CA-3′ and 5′-TGC GTT AGC TAC ACT GTG TTC-3′), respectively. When necessary, a second-round PCR, using inner primers E6/E7 (5′-TTA CCG TAC CCA CAA CCA CT-3′ and 5′-CCT CTA CTT CAA ACC AGC CT-3′), L1A (5′-GGT CCT GAC ATT CCA TTA CC-3′ and 5′-GGG CAC ATC ACT TTT ACT AGC-3′), L1B (5′-ACA GGA TTT GGT TGC ATG G-3′ and 5′-TTC TTT GTG GAG GTA CGT GG-3′) and LCR (5′-TTT GTT ACA GGC AGG GCT AC-3′ and 5′-CGT TTT CGG TTA CAC CCT A-3′), was performed **(**S2 Table). Each PCR was conducted in a 30-μL reaction mixture containing 0.75 unit of HotStarTaq *Plus* DNA Polymerase (QIAGEN, Hilden, Germany), 200 μM of dNTPs and 0.25 μM of each primer. An aliquot of 3 μL of extracted DNA was added as template. The thermal cycling began with a 5-min initial denaturation and enzyme activation at 95°C, followed by 35 cycles of 1-min denaturation at 94°C, 1-min annealing at 58°C and 40-sec extension at 72°C, and ended with an 8-min final extension at 72°C. PCR products were sequenced from both directions and analyzed using Seq-Scape software (version 2.5, Applied Biosystems, Foster City, CA, USA). Repeated sequencing was performed as a confirmation when mutations occurred only once.

### Phylogenetic tree construction

A maximum-likelihood tree was constructed using MEGA6 (Molecular Evolutionary Genetic Analysis software program, version 6.0; http://www.megasoftware.net) [13]. The tree was comprised of concatenated E6-E7-L1-LCR sequences of unique HPV52 strains collected in this study and from a published reference strain of each lineage (A1: X74481, A2: HQ537739, B1: HQ537740, B2: HQ537743, C1: HQ537744, C2: HQ537746, D: HQ537748). Bootstrap values of key nodes were generated by 1000 resamplings. To root the tree, HPV67 prototype sequences (NCBI accession no. NC_004710) were set as the outgroup.

### Statistical analysis

Statistical analysis was performed using a commercially available statistical software package (SPSS statistical software version 18.0 [SPSS Inc, Chicago, IL, USA]). Fisher’s exact test and logistic regression analysis were used to analyze categorical data. The level of significance was set at *P* < 0.05.

## Results

### Characteristics of cervical samples

Of the 91 cervical cytology samples, 40 (44.0%) showed normal pathologic findings (normal squamous cell epithelium; Table 1). We separated pre-malignant lesions into ‘low-grade lesions’ and ‘high-grade lesions’. ‘Low-grade lesions’ (n = 22) included ASCUS (atypical squamous cells of undetermined significance) and LGSIL (low-grade squamous intraepithelial lesions), while ‘high-grade lesions’ (n = 21) included HGSIL (high-grade squamous intraepithelial lesions), CIN3 (cervical intraepithelial neoplasia 3) and CIS (carcinoma *in situ*). For malignant lesions, seven (7.7%) squamous cell carcinomas (SCCs) were included.

### Lineage identification

**Fig 1:**
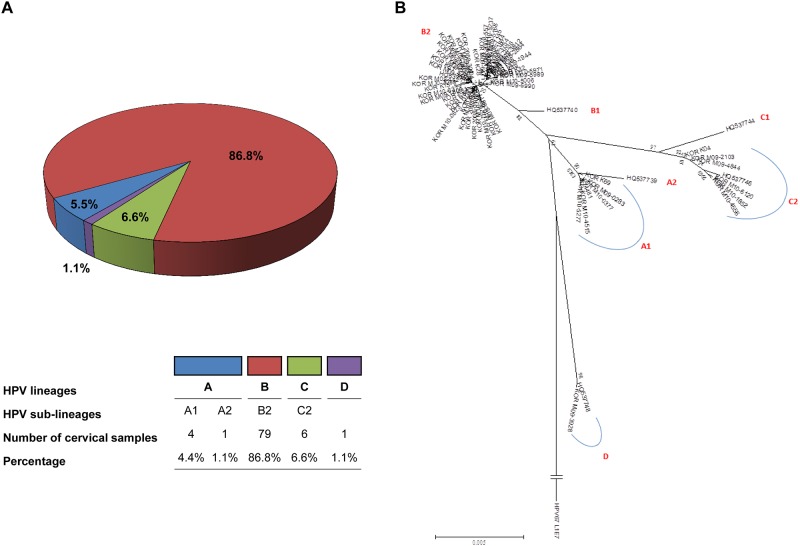
HPV52 variant lineage distribution of study samples. (A) Lineages A (sublineages: A1 and A2), B (sublineage: B2), C (sublineage: C2) and D were detected. (B) A phylogenetic tree was constructed from 57 HPV52 variants using concatenated L1, LCR, E6 and E7. A maximum-likelihood tree was constructed using the program, MEGA6. Bootstrap values of key nodes generated by 1,000 resamplings are shown. The length of the scale bar represents 0.005 substitutions per nucleotide position. To root the tree, HPV67 prototype sequences (NCBI accession no. NC_004710) were set as outgroup. The GenBank accession no. of study samples are KY077824-KY077901.

Altogether, four HPV52 variant lineages were identified based on the phylogenetic tree topology (Fig 1**)**. Lineages A, B and C were closely related, while lineage D was relatively distant. Lineage B was most frequently detected (86.8%, 79 of 91 samples; Table 1). The majority of high-grade lesions (80.9%, 17 of 21 samples) belonged to lineage B, and the remaining high-grade lesions belonged to lineage C (19.1%). Lineage B harbored all of the seven SCCs and their association was significant (*P* = 0.02).

### HPV52 sequence variations

In this study, 40.6% (3226 nucleotides) of the HPV52 genome (7942bps, X74481) was sequenced (S3–S6 Tables). Nine E6 variants with 11 nucleotide positions showing sequence polymorphisms were identified, encompassing three nonsynonymous mutations (S3 Table). Five variants with 12 nucleotide positions showing sequence polymorphisms with seven nonsynonymous mutations were identified in E7 (S4 Table). L1 harbored 26 variants and 54 nucleotide sequence polymorphisms with 11 nonsynonymous mutations (S5 Table). The noncoding LCR was the most heterogeneous, encompassing 38 variants showing 87 nucleotide sequence variations **(**S6 Table**)**.

An analysis of sequences for the E6, E7, L1 and LCR genes suggested that HPV52 harbors lineage-specific variations. Lineage B, the most frequently detected lineage, harbored a number of lineage-specific variations (Table 2). Of the 91 samples, K93R (A379G), the most frequently detected nonsynonymous mutation (85.71%) in E6, was only found in lineage B. In addition, a novel mutation, 7935_7936 insT in LCR was significantly associated with lineage B (*P* < 0.0001). Lineage C also harbored lineage-specific variations (Table 3), of which an E6 nonsynonymous mutation (L83V, concurrent mutations of C348G and G350T) and five E7 nonsynonymous mutations, S52D (concurrent mutations of A706G and G707A), Y55D (T727G), H61Y (C733T), D64N (G742A) and L99R (T848G), showed significant associations with the lineage (*P* < 0.0001).

**Table 2:** List of nucleotide variations associated with HPV52 lineage B.

		Non-B lineages (n = 12)	B lineage (n = 79)	*P* value*	Adjust odds ratio^†^ (95% CI)	*P* value^†^
E6 nucleotide change						
	G350T	7	79	<0.0001	77.31 (12.39 –Infinity)	<0.0001
	A379G	0	78	<0.0001	186.39 (48.04 –Infinity)	<0.0001
E7 nucleotide change						
	C751T	0	79	<0.0001	92.87 (35.35 –Infinity)	<0.0001
	A801G	7	79	<0.0001	77.31 (12.39 –Infinity)	<0.0001
L1 nucleotide change						
	A5771G	0	76	<0.0001	350.56 (59.44–Infinity)	<0.0001
	T5972C	0	78	<0.0001	186.39 (48.04– Infinity)	<0.0001
	G6110A	0	79	<0.0001	92.87 (35.35–Infinity)	<0.0001
	G6218A	7	79	<0.0001	77.31 (12.39–Infinity)	<0.0001
	T6710G	0	79	<0.0001	92.87 (35.35–Infinity)	<0.0001
	T6764C	0	79	<0.0001	92.87 (35.35–Infinity)	<0.0001
	A6794G	0	78	<0.0001	186.25 (47.99–Infinity)	<0.0001
	C6824T	0	79	<0.0001	92.87 (35.35–Infinity)	<0.0001
	C6917A	7	78	<0.0001	84.04 (7.25–974.61)	0.0004
	G7052A	2	78	<0.0001	166.80 (21.96–Infinity)	<0.0001
LCR nucleotide change						
	G7168C	6	71	0.0025	8.89 (2.31–34.20)	0.0015
	C7207A	7	73	0.0051	9.32 (2.17–40.10)	0.0027
	G7371T	4	77	<0.0001	171.31 (14.34–Infinity)	<0.0001
	G7622A	7	79	<0.0001	77.31 (12.39–Infinity)	<0.0001
	T7624G	7	79	<0.0001	77.31 (12.39–Infinity)	<0.0001
	A7657C	0	79	<0.0001	92.87 (35.35–Infinity)	<0.0001
	T7659C	6	79	<0.0001	105.84 (17.45–Infinity)	<0.0001
	G7712C	7	79	<0.0001	77.31 (12.39–Infinity)	<0.0001
	G7861A	7	79	<0.0001	77.31 (12.39–Infinity)	<0.0001
	A7865G	1	77	<0.0001	225.24 (41.70–Infinity)	<0.0001
	7935_7936 insT	7	79	<0.0001	77.31 (12.39–Infinity)	<0.0001
	A7938G	1	48	<0.0001	18.05 (2.19–148.62)	0.0071
	T13C	0	79	<0.0001	92.87 (35.35–Infinity)	<0.0001

Statistical analyses were performed using *Fisher's exact test, ^†^multivariable logistic regression (age-adjusted).

**Table 3:** List of nucleotide variations associated with HPV52 lineage C.

		Non-C lineages (n = 85)	C lineage (n = 6)	*P* value*	Adjust odds ratio^†^ (95% CI)	*P* value^†^
E6 nucleotide change						
	A530G	0	6	<0.0001	41.78 (14.64–Infinity)	<0.0001
	C348G and G350T	0	3	<0.0001	52.83 (7.31–Infinity)	0.0011
E7 nucleotide change						
	T573A	0	6	<0.0001	41.78 (14.64–Infinity)	<0.0001
	C662T	0	6	<0.0001	41.78 (14.64–Infinity)	<0.0001
	A706G and G707A	0	6	<0.0001	41.78 (14.64–Infinity)	<0.0001
	T727G	0	6	<0.0001	41.78 (14.64–Infinity)	<0.0001
	C733T	0	6	<0.0001	41.78 (14.64–Infinity)	<0.0001
	G742A	0	6	<0.0001	41.78 (14.64–Infinity)	<0.0001
	T848G	0	6	<0.0001	41.78 (14.64–Infinity)	<0.0001
L1 nucleotide change						
	T5578C	0	3	<0.0001	140.86 (16.81–Infinity)	0.0001
	G5720A	0	3	<0.0001	52.83 (7.31–Infinity)	0.0011
	A5909G	0	5	<0.0001	155.81 (26.17–Infinity)	<0.0001
	G6083A	0	6	<0.0001	41.78 (14.64–Infinity)	<0.0001
	C6443T	0	6	<0.0001	41.78 (14.64–Infinity)	<0.0001
	G6698A	0	6	<0.0001	41.78 (14.64–Infinity)	<0.0001
	G7112A	0	6	<0.0001	41.78 (14.64–Infinity)	<0.0001

Statistical analyses were performed using *Fisher's exact test, ^†^multivariable logistic regression (age-adjusted).

## Discussion

HPVs are circular double-stranded DNA viruses that consist of heterogeneous variants with different pathogenicities [4, 14]. HPV52 is one of the most frequently detected carcinogenic high-risk genotypes in East Asia [15–17] and is one of the genotypes targeted by the recent, USA FDA-approved HPV 9-valent vaccine [9]. However, only a few studies on the pathogenicity of HPV52 are available [18]. In this regard, we attempted to achieve three aims. Firstly, we aimed at identifying HPV52 variants and lineages based on E6, E7, L1 and LCR sequences. Secondly, we attempted to delineate lineage-specific variations. Lastly, we sought to examine the risk association of various lineages, variants and mutations. As a result, we identified four HPV52 variant lineages (A, B, C and D), with lineage B (86.8%) being the most frequently detected, followed by lineages C (6.6%), A (5.5%), and D (1.1%). By further analyzing the two most frequently detected lineages, we found distinct sequence variations in each lineage. Of note, one of lineage B-specific mutations was found to associate with a higher risk for cervical cancer.

Our findings of the two most frequently detected lineages in HPV52, B and C, also confirm previous observations [19, 20]. These two lineages are associated with high-grade lesions [19, 21], and we have shown that they harbor a number of lineage-specific variations (Tables 2 and 3). Lineage B-specific mutations included the most frequently detected nonsynonymous mutation K93R (A379G) in E6, while lineage C-specific mutations included an E6 nonsynonymous mutation (L83V, concurrent mutations of C348G and G350T) and five E7 nonsynonymous mutations, S52D (concurrent mutations of A706G and G707A), Y55D (T727G), H61Y (C733T), D64N (G742A) and L99R (T848G)).

Our findings suggest that lineage-specific mutations may contribute to the carcinogenicity of each HPV52 lineage. E6 is an oncogene that interacts with a well-known tumor suppressor, *TP53*, increasing the risk for the accumulation of genetic changes [22] and inhibiting cellular responses such as cell cycle arrest, induction of apoptosis and DNA damage repair [23, 24]. K93R (A379G) is a nonsynonymous mutation located in the E6 oncogene, which may have a specific role in carcinogenesis: it is not only the most frequently detected variation [25], but is also independently associated with high-grade lesions [26]. An alteration at nucleotide position 350 in E6 was found for both lineages B and C. However, lineage C harbored an additional alteration at nucleotide position 348, yielding a nonsynonymous mutation, L83V. L83V have been reported not only in HPV52, but also in HPV16 and HPV33 [25, 27, 28]. In addition, an association of HPV16 L83V with high-grade lesions has been shown [29], suggesting that it may contribute to the pathogenicity of lineage C [19]. It is noticeable that E7 nonsynonymous mutations (S52D, 55D, H61Y, D64N and L99R) were harbored by lineage C, but not by lineage B. E7 interacts with the retinoblastoma (Rb) protein, causing uncontrolled cell division and inactivating its function as a tumor suppressor [30, 31].

## Conclusions

HPV52 is the second to fifth most frequently detected high-risk HPV genotype in Korea [15, 32–34]. Our data demonstrated for the first time that HPV52 lineages (B and C) circulating in Korea harbor distinct genetic alterations that may affect pathogenicity. We also observed that most of the cervical samples (ranging from normal cervix to SCC) were infected with HPV52 lineages B or C that carried putative high-risk mutations. Our findings may provide a useful basis to understand the heterogeneity of HPV52 variants in Korea, and to assist the development of diagnostic assays and vaccines.

## Supporting Information

S1 Table Primers for HPV-52 long-fragment PCR amplification. (XLSX)

S2 Table Primers for HPV-52 short-fragment PCR amplification. (XLSX)

S3 Table Nucleotide sequence variations of HPV52 E6. (XLSX)

S4 Table Nucleotide sequence variations of HPV52 E7. (XLSX)

S5 Table Nucleotide sequence variations of HPV52 L1. (XLSX)

S6 Table Nucleotide sequence variations of HPV52 LCR. (XLSX)

## Abbreviations

ASCUS: atypical squamous cells of undetermined significance
CIN3: cervical intraepithelial neoplasia 3
CIS: carcinoma *in situ*
HGSIL: high-grade squamous intraepithelial lesions
HPV: Human papillomavirus
LGSIL: low-grade squamous intraepithelial lesions
SCC: squamous cell carcinoma
